# Glutathione S-transferase M1 and T1 genes deletion polymorphisms and risk of developing essential hypertension: a case-control study in Burkina Faso population (West Africa)

**DOI:** 10.1186/s12881-020-0990-9

**Published:** 2020-03-19

**Authors:** Herman Karim Sombié, Abel Pegdwendé Sorgho, Jonas Koudougou Kologo, Abdoul Karim Ouattara, Sakinata Yaméogo, Albert Théophane Yonli, Florencia Wendkuuni Djigma, Daméhan Tchelougou, Dogfounianalo Somda, Isabelle Touwendpoulimdé Kiendrébéogo, Prosper Bado, Bolni Marius Nagalo, Youssoufou Nagabila, Enagnon Tiémoko Herman Donald Adoko, Patrice Zabsonré, Hassanata Millogo, Jacques Simporé

**Affiliations:** 1 Laboratory of Molecular Biology and Genetics (LABIOGENE), UFR/SVT, University Joseph Ki-Zerbo, 03 P.O. Box 7021, Ouagadougou 03, Burkina Faso; 2 Saint Camille Hospital of Ouagadougou (HOSCO), 01 P.O. Box 444, Ouagadougou 01, Burkina Faso; 3 University Hospital Center-Yalgado Ouédraogo (CHUYO), 01 P.O. Box 676, Ouagadougou, Burkina Faso; 4 Pietro Annigoni Biomolecular Research Center (CERBA), P.O. Box 364, Ouagadougou 01, Burkina Faso; 5 Faculty of Medicine, University Saint Thomas d’Aquin, P.O. Box 10212, Ouagadougou, Burkina Faso

**Keywords:** Essential hypertension, *GSTM1*, *GSTT1*, Null genotypes, Burkina Faso

## Abstract

**Background:**

Glutathione S-transferases play a key role in the detoxification of persistent oxidative stress products which are one of several risks factors that may be associated with many types of disease processes such as cancer, diabetes, and hypertension. In the present study, we characterize the null genotypes of *GSTM1* and *GSTT1* in order to investigate the association between them and the risk of developing essential hypertension.

**Methods:**

We conducted a case-control study in Burkina Faso, including 245 subjects with essential hypertension as case and 269 control subjects with normal blood pressure. Presence of the *GSTT1* and *GSTM1* was determined using conventional multiplex polymerase chain reaction followed by gel electrophoresis analysis. Biochemical parameters were measured using chemistry analyzer CYANExpert 130.

**Results:**

Chi-squared test shows that *GSTT1-null* (OR = 1.82; *p* = 0.001) and *GSTM1-active/GSTT1-null* genotypes (OR = 2.33; *p* <  0.001) were significantly higher in cases than controls; the differences were not significant for *GSTM1*-*null*, *GSTM1*-*null*/*GSTT1-active* and *GSTM1-null*/*GSTT1-null* (*p* > 0.05). Multinomial logistic regression revealed that age ≥ 50 years, central obesity, family history of hypertension, obesity, alcohol intake and *GSTT1* deletion were in decreasing order independent risk factors for essential hypertension. Analysis by gender, BMI and alcohol showed that association of *GSTT1*-null with risk of essential hypertension seems to be significant when BMI < 30 Kg/m^2^, in non-smokers and in alcohol users (all OR ≥ 1.77; *p* ≤ 0.008). Concerning *GSTT1*, *GSTM1* and cardiovascular risk markers levels in hypertensive group, we found that subjects with *GSTT1-null* genotype had higher waist circumference and higher HDL cholesterol level than those with *GSTT1-active* (all *p* <  0.005), subjects with *GSTM1-null* genotype had lower triglyceride than those with *GSTM1*-*active* (*p* = 0.02) and subjects with the double deletion *GSTM1-null*/*GSTT1-null* had higher body mass index, higher waist circumference and higher HDL cholesterol than those with *GSTM1*-*active*/*GSTT1*-*active* genotype (all *p* = 0.01).

**Conclusion:**

Our results confirm that *GSTT1-null* genotype is significantly associated with risk of developing essential hypertension in Burkinabe, especially when BMI < 30 Kg/m^2^, in non-smokers and in alcohol users, and it showed that the double deletion *GSTM1-null*/*GSTT1-null* genotypes may influence body lipids repartition.

## Background

High blood pressure is the most common risk factor for cardiovascular mortality and morbidity worldwide and is thought to be responsible for just under 8 million deaths a year worldwide and nearly 100 million days of disability [1, 2]. In 2000, the number of adults with hypertension in sub-Saharan Africa was estimated at 80 million [3] and in Burkina Faso, more than 18% of the adult population had hypertension in 2013 [4]. High blood pressure is a complex pathology resulting from the interactions of multiple genetic and environmental determinants. It is described in ≈ 90% of cases as essential hypertension when the precise causes of the disease remain poorly understood [5, 6].

There is considerable evidence that oxidative stress resulting from an imbalance between the generation of reactive oxygen species (ROS) and the body’s antioxidant defense systems is involved in the pathophysiology of hypertension [7–13]. Several studies have shown that subjects with hypertension and several animal models of hypertension produce an excessive amount of reactive oxygen species [9, 10] and have abnormal levels of antioxidant [13]. Other studies have shown that the blood pressure of mouse models that are genetically deficient in reactive oxygen species generating enzymes is lower than that of their wild-type counterparts [12]. In cultured vascular smooth muscle cells and arteries isolated from hypertensive and hypertensive rats, the production of reactive oxygen species is increased, the redox-dependent signaling is amplified and the bioactivity of the antioxidants is reduced [11]. In addition, there is some evidence to suggest that reactive oxygen species play a key role in the pathophysiological thought to be the cause of hypertension [7, 8]. Reactive oxygen species are highly reactive atoms or molecules with one or more unpaired electron(s) in their external shell such as superoxide anion, hydrogen peroxide, and hydroxyl radical formed by the partial reduction of oxygen [14]. Reactive oxygen species have endogenous sources as in the process of mitochondrial oxidative phosphorylation and exogenous sources such as air and water pollution, tobacco, alcohol, heavy or transition metals, drugs, industrial solvents, cooking and radiation, which inside the body are metabolized into free radicals [15]. GSTs are an enzyme superfamily that plays a central role in Phase II of the cellular detoxification process of a diverse group of exogenous and endogenous harmful compounds [16]. They combine reduced glutathione (GSH) to produce a wide variety of lipophilic compounds and having an electrophilic center [17]. This reaction gives a GSH conjugate, often inactive, soluble in water and generally less toxic than the parent compounds such as phenols, plant aflatoxins, superoxide radical and hydrogen peroxide [18]. In addition to conventional conjugation reactions, GSTs exhibit glutathione peroxidase activity and catalyze the reduction of organic hydroperoxides such as phospholipids, fatty acids and DNA hydroperoxides to their corresponding alcohols (Hayes et al., 2005). They are also involved in the modulation of signal transduction pathways involved in cell survival and apoptosis, where they control the activity of mitogen-activated protein kinase (MAPK) members [19]. Eight distinct classes of soluble GST in cytoplasmic mammals have been identified named *GSTA, GSTM, GSTT, GSTP, GSTS, GSTK, GSTO*, *GSTZ* [20] and Two loci in particular Glutathione S-transferases Mu 1 (*GSTM1*) and Theta 1 (*GSTT1*) are the most studied. In human, a significant number of genetic polymorphisms among the GST have been described [16] and individual differences in GST activity are the result of genetic polymorphisms. The most common variant of *GSTM1* and *GSTT1* genes is homozygous deletion (null genotype) which has been associated with loss of enzymatic activity, oxidative damage and increased vulnerability to cytogenetic [21, 22].

Several cases-controls studies have reported that *GSTT1* and/or *GSTM1* null genotype were associated to the risk of developing hypertension in some populations, but rather the results are still controversial [23–31]. In this study, we aimed to characterize firstly null variants of *GSTM1* and *GSTT1* in Burkina Faso and secondly to evaluate the association between them and the risk of developing essential hypertension.

## Methods

### Study design

The Internal Research Ethics Committee of CERBA/LABIOGENE and National Ethics Committee for Health Research of Burkina Faso approved the protocol of this case-control study. We recruited a total of 514 subjects, including 245 patients with essential hypertension and 269 subjects with normal blood pressure in the cardiology and general consultation departments at Saint Camille Hospital in Ouagadougou and at the Yalgado Ouédraogo University Hospital Center in Ouagadougou. All participants were from the central region of Burkina Faso and resided there.

Essential hypertension was determined by the cardiologist when no secondary cause of blood pressure elevation was present [32]. Subjects with systolic blood pressure less than 130 mmHg and diastolic blood pressure below 80 mmHg, without history of hypertension, and who are not on antihypertensive therapy, were selected as normotensive controls.

### Samples and data collection

When a subject met the above selection criteria, he was referred by the cardiologist to the principal investigator who was responsible for clearly explaining the study. Once his consent obtained in a free and transparent manner, using a standardized questionnaire completed throughout the study, data on anthropometric parameters, lifestyle, clinical and biological parameters were collected (see questionnaire in ***Supplementary file*** ***3***). The information collected mainly were age, sex, parents’ ethnicity, occupation, weight, height, waist circumference, lifestyle, blood pressure, personal, family history, electrocardiogram data, cardiac echo-Doppler data and biochemical data.

Blood pressure measurements were performed using a manual aneroid sphygmomanometer and electronic cuffed sphygmomanometer by the cardiologist and the principal investigator. Blood pressure was taken on both arms in a sitting position after at least 20 min of rest. All measurements were made at least twice with a minimum of 5 min between two measurements. Blood pressure values ​​were obtained by averaging the measurements.

Height and weight were measured and body mass index (BMI) was calculated by dividing the weight (Kilogram) by the square of the height (meters). BMI was used to determine obesity when BMI ≥ 30 kg/m^2^, overweight when BMI was between 25 and 30 kg/m^2^, normal weight when BMI was between 20 and 25 kg/m^2^ and underweight for a BMI less than 20 kg/m^2^.

Waist circumference (WC) was determined by measuring the circumference of the abdomen when the subject has minimal breathing using a tape measure. Abdominal obesity was determined in men when WC is greater than 102 cm and in women when it is greater than 88 cm [33].

Participants with a family history of hypertension were defined as those with at least one close family member hypertensive before the age of 60 years.

Each participant also had a venous blood sample of approximately 8 ml in two tubes (EDTA and tube without anticoagulant) for analysis. Sera from anticoagulant-free tubes were directly used for biochemical analyzes in the biochemistry laboratory (CYANExpert 130), and blood white cells from EDTA tubes were placed in cryotubes and stored at − 20 °C in the molecular biology laboratory until extraction of the DNA.

### DNA extraction and genotyping

We used standard salt fractionation method as described by Miller *and al.* in 1988, to isolated genomic DNA from peripheral blood white cells [34]. The purity and concentration of the resulting DNA were determined using Biodrop μLITE (Isogen Life Science, Temse, Belgium) and the extracts were stored at − 20 °C until use.

The presence (homozygous +/+ and heterozygous +/−) or absence (homozygous for deletion −/−) of the *GSTM1* and *GSTT1* genes has been determined according to the method described by Chen *and al* [35]. Briefly we performed multiplex PCR with the GeneAmp PCR system 9700 (Applied Biosystem, USA) in a reaction volume of 25 μL including 10 μL of Master Mix Ampli Taq Gold® (Applied Biosystems, USA), 1 μL of each of the primer pairs of each gene obtained from Biosynthesis, 7 μL of nuclease-free water and 5 μL of DNA. *GSTM1* primers were (forward- 5′ GAA CTC CCT GAA AAG CTA AAG C 3′ and reverse- 5′ GTT GGG CTC AAA TAT ACG GTG G 3′), *GSTT1* primers were (forward- 5′ TTC CTT ACT GGT CCT CAC ATC TC 3′ and reverse- 5′ TCA CCG GAT CAT GGC CAG CA 3′) and *β-globin* primers were (forward- 5’CAA CTT CAT CCA CGT TCA CC 3′ and reverse- 5′ GAA GAG CCA AGG ACA GGT AC 3′). All reagents were purchased from Applied Biosystems (ABI, Applera International Inc., Foster City, CA, USA). The amplification program was as follows: an activation phase at 94 °C for 5 min; 40 cycles of a series of denaturation at 94 °C for 1 min, hybridization at 57 °C for 1 min, elongation at 72 °C for 1 min; and a final extension at 72°c for 7 min. PCR products undergo at ethidium bromide-stained 3% agarose gel migration during 45 mn and visualized under UV light at 312 nm using the Geneflash revelation device. Samples in which the *GSTM1*, *GSTT1* and *β-globin* genes are present yielded 219-bp, 480-bp and 268-bp product respectively; the absence amplifiable *GSTM1* or *GSTT1* indicates the respective null genotype for each. PCR amplification was considered valid if the sample had a band corresponding to that of *β-globin*. ***Supplementary Fig.*** ***1*****.**

### Statistical analysis

Data analyses were performed by using Statistical Package for Social Sciences (SPSS Version 20.0) and Epi Info (Version 6.0).

Following values have been taken into account to determinate sample size using Epi Info Version 6.0: 95% of two-sided confidence level, 80% of power, odds ratio more than 1.7 ratio of controls to cases 1.1, the proportion of control group having null genotypes of *GSTM1* and *GSTT1* about 30%.

We expressed quantitative variables as mean ± standard deviation and comparison between groups was assessed with Student’s t-test.

Genotypic frequencies were expressed as percentage and comparisons between cases and controls were done with the chi-squared test.

To research factors associated with risk of essential hypertension in our study and possible interactions between them, we performed a multinomial logistic regression analysis (forward stepwise method) by considering hypertensive status as a dependent variable and including the factors we thought were involved in the development of essential hypertension.

For all analyses, difference was statistically significant when *p* <  0.05.

## Results

### Quantitative characteristics

The characteristics of the study population are given in Table 1. We included a case group of 245 subjects with a diagnosis of essential hypertension (111 males and 134 females; 50.14 ± 8.22 years old) and a control group of 269 normotensive individuals (129 males and 140 females; 48.69 ± 9.43 years old). Statistical analysis of the distribution by sex and means of age showed no significant differences between cases and controls (*p* > 0.05), indicating that there is homogeneity between groups.

**Table 1:** General Characteristics of the study population

Parameters	Total, *n* (%) 514 (100%)	Cases, *n* (%) 245 (100%)	Controls, *n* (%) 269 (100%)	*p* value
Gender (M/F)	240/274	111/134	129/140	0.59
Age (years)	49.38 ± 8.90	50.14 ± 8.22	48.69 ± 9.43	0.06
SBP (mmHg)	139.27 ± 30.32	166.24 ± 19.92	114.71 ± 11.35	< 0.001*
DBP (mmHg)	83.77 ± 16.56	97.28 ± 12.09	71.47 ± 8.55	< 0.001*
BMI (Kg/m^2^)	25.98 ± 6.19	28.35 ± 6.40	23.82 ± 5.11	< 0.001*
WC (cm)	90.28 ± 12.21	96.28 ± 12.26	84.81 ± 9.25	< 0.001*
Fasting Blood Glucose (mM)	5.15 ± 1.81	5.69 ± 1.56	3.75 ± 1.69	< 0.001*
HDL-c (mM)	1.31 ± 0.60	1.40 ± 0.63	1.07 ± 0.42	0.001*
LDL-c (mM)	2.90 ± 1.12	3.02 ± 1.09	2.61 ± 1.14	0.04*
Total Cholesterol (mM)	4.90 ± 1.29	5.10 ± 1.18	4.38 ± 1.44	0.001*
Triglycerides (mM)	1.18 ± 0.75	1.25 ± 0.77	1.00 ± 0.65	0.51
Creatinine (μM)	98.40 ± 23.82	96.80 ± 25.54	106.10 ± 10.38	0.26
Calcium (mM)	2.54 ± 1.04	2.51 ± 1.09	2.68 ± 0.83	0.63
Magnesium (mM)	0.79 ± 0.29	0.81 ± 0.31	0. 69 ± 0.11	0.22
Sodium (mM)	140.46 ± 5.47	139.98 ± 5.38	142.54 ± 5.62	0.16
Potassium (mM)	4.16 ± 0.82	4.01 ± 0.71	4.80 ± 1.00	0.03*
Chlorine (mM)	102.78 ± 5.99	102.24 ± 5.64	105.17 ± 7.14	0.14

Values are reported as means ± standard deviation for continuous variables; Statistical analysis (Cases versus controls) by t test or chi-square; *: significant difference between groups (*p* < 0.05); *MD* Means difference, *CI* Confidence interval, *SBP* Systolic blood pressure, *DBP* Diastolic blood pressure, *WC* Waist circumference, *HDL-c* High density lipoprotein cholesterol, *LDL-c* Low density lipoprotein cholesterol, *mM* Millimolar, *μM* Micromolar

We found that means of body mass index, waist circumference, serum levels of blood sugar, Total cholesterol, LDL Cholesterol, HDL cholesterol were higher in hypertensive compared to normotensive group and differences were significant (all *p* <  0.05). These results suggest that many of our patients with essential hypertension are overweight or obese, and that many may also have diabetes and / or hypercholesterolemia, although it is difficult for us to confirm this based on our unique biochemical assay.

We also found that serum levels of Potassium was higher in normotensive group compared to hypertensive (*p* = 0.03), but there was no significant difference in level of Triglycerides, creatinine, Calcium, Sodium, Magnesium and Chlorine between the two groups (all *p* > 0.05).

### Genetics analysis

The Table 2 shows the distribution of *GSTM1* and *GSTT1* variants in the study population. A total of 514 subjects (245 cases and 269 controls) were genotyped for the deletion genotype of two GST isoforms. In the general study population, we found that the frequency of *GSTM1-active* and *GSTT1-active* were 71.60 and 37.94% respectively; those of *GSTM1-null* and *GSTT1-null* were 28.40 and 62.06% respectively. Based on the ratio controls to cases, odds ratio, and frequencies of *GSTM1*-null and *GSTT1*-null, the genetic power calculator indicated that the sample size is large enough to perform a case-control analysis with 85% power for *GSTT1*, those of *GSTM1* is less than 40%.

**Table 2:** Distribution of genotypic frequencies for *GSTM1* and *GSTT1* in the study population

Genotypes	Total *n* (%) 514 (100%)	Cases *n* (%) 245 (100%)	Controls *n* (%) 269 (100%)	OR	CI 95%	*p* value
*# GSTM1-active* (+)	368 (71.60)	183 (74.69)	185 (68.77)	1.00 *(reference group)*		
*GSTM1-null* (−)	146 (28.40)	62 (25.30)	84 (31.23)	0.74	0.50–1.09	0.14
*# GSTT1-active* (+)	194 (37.74)	75 (30.61)	119 (44.24)	1.00 *(reference group)*		
*GSTT1-null* (−)	320 (62.26)	170 (69.39)	150 (55.76)	1.79	1.25–2.58	0.001*
*# GSTM1*(*+*) */ GSTT1*(*+*)	125 (24.32)	45 (18.37)	80 (29.74)	1.00 *(reference group)*		
*GSTM1*(*−*) */ GSTT1*(*+*)	69 (13.42)	30 (12.25)	39 (14.50)	1.36	0.75–2.49	0.35
*GSTM1*(*+*) */ GSTT1*(*−*)	243 (47.28)	138 (56.32)	105 (39.03)	2.33	1.50–3.65	< 0.001*
*GSTM1*(*−*) */ GSTT1*(*−*)	77 (14.98)	32 (13.06)	45 (16.73)	1.26	0.71–2.26	0.45

Analysis by chi-square to obtain odds ratio values (OR) and confidence interval; (+): active; (−): null; *CI* Confidence interval, *OR* Odds ratio; #: reference; *: significant difference between groups (*p* < 0.05)

When we compared these frequencies between cases and controls, we found that subjects with *GSTT1-null* genotype (69.39% versus 55.39%; OR = 1.82; *p* = 0.001) and *GSTM1-active/GSTT1-null* genotype (56.32% versus 39.03%; OR = 2.33; *p* <  0.001) were more present in case group than controls and difference between the two group was significant. But we didn’t find a significant difference between cases and controls concerning *GSTM1*-*null* genotype (25.30% versus 31.23%; OR = 0.74; *p* = 0.14) and the double deletion *GSTM1-null*/*GSTT1-null* (13.06% versus 16.73%; OR = 1.26; *p* = 0.45).

The Table 3 presents multinomial logistic regression for essential hypertension risk factors in our study population to obtain adjusted odds ratio values and confidence intervals. We found that advanced age (≥ 50 years; OR = 5.33; *p* <  0.001), central obesity (OR = 4.80; *p* <  0.001), family history of hypertension (OR = 4.61; *p* <  0.001), obesity (OR = 3.95; *p* = 0.001), alcohol intake (OR = 2.16; *p* <  0.001) and *GSTT1* deletion (OR = 1.81; *p* = 0.001) were in decreasing order independent risk factors for developing essential hypertension in our general study population.

**Table 3:** Multinomial logistic regression for risk analysis of essential hypertension

Factors	OR	CI 95%	*p* value
Obesity	3.95	2.48–6.29	0.001*
Central obesity	4.80	3.23–7.14	< 0.001*
Alcohol intake	2.16	1.5–3.1	< 0.001*
Smoking	1.24	0.49–3.16	0.8
Sex M	0.89	0.63–1.27	0.59
Family history of HTA	4.61	3.17–6.69	< 0.001*
Age ≥ 50 years	5.33	3.61–7.86	< 0.001*
*GSTT1-*null	1.81	1.26–2.60	0.001*

*Significant difference between groups (*p* < 0.05); *CI*, Confidence interval, *OR* Odds ratio

Considering BMI, alcohol and smoking difference in essential hypertension, we further stratified genotyping results by BMI, smoking and alcohol status (Table 4**).** Interestingly results showed that association between *GSTT1*-null and essential hypertension seems to be significant when BMI < 30 Kg/m^2^ (OR = 1.77; *p* = 0.008), in non-smokers (OR = 1.86; *p* = 0.003) and in alcohol users (OR = 2.26; *p* = 0.007).

**Table 4:** BMI, age, and sex stratified analysis of association between *GSTM1* and *GSTT1* variants with essential hypertension

Genes	Parameters	groups	Variants (*n*)		OR	CI (95%)	*p* value
				active			
*GSTM1*	BMI < 30 Kg/m^2^	Cases	38	124	–		
		Controls	72	167	0.71	0.45–1.12	0.17
	BMI ≥ 30 Kg/m^2^	Cases	24	59	–		
		Controls	12	18	0.61	0.25–1.45	0.36
*GSTT1*	BMI < 30 Kg/m^2^	Cases	113	49	–		
		Controls	135	104	1.77	1.16–2.70	0.008*
	BMI ≥ 30 Kg/m^2^	Cases	57	26	–		
		Controls	15	15	2.19	0.93–5.14	0.07
*GSTM1*	Smoking YES	Cases	15	50	–		
		Controls	13	48	1.10	0.47–2.57	0.83
	Smoking NO	Cases	47	133	–		
		Controls	71	137	0.68	0.43–1.05	0.09
*GSTT1*	Smoking YES	Cases	46	19	–		
		Controls	37	24	1.57	0.74–3.29	0.26
	Smoking NO	Cases	124	56	–		
		Controls	113	95	1.86	1.22–2.82	0.003*
*GSTM1*	Alcohol intake YES	Cases	26	92	–		
		Controls	27	52	0.47	0.25–0.88	0.07
	Alcohol intake NO	Cases	36	91	–		
		Controls	57	133	0.99	0.60–1.63	0.80
*GSTT1*	Alcohol intake YES	Cases	86	32	–		
		Controls	45	38	2.26	1.25–4.10	0.007*
	Alcohol intake NO	Cases	84	43	–		
		Controls	105	81	1.50	0.94–2.40	0.09

Analysis by chi-square to obtain odds ratio values (OR) and confidence interval; *CI* Confidence interval, *OR* Odds ratio; *: significant difference between groups (*p* < 0.05)

In addition, concerning *GSTT1*, *GSTM1* and cardiovascular risk markers levels in hypertensive group we compared the average of cardiovascular risk markers between the *GSTM1* and *GSTT1* null and active genotypes. We found that hypertensive individuals with *GSTM1-null* genotype had a lower average triglyceride than *GSTM1-active* genotypes; *GSTT1-null* genotype had a higher average waist circumference and HDL cholesterol than *GSTT1-active* genotype and the double deletions *GSTM1-null/GSTT1-null* have higher body mass index, higher waist circumference and higher HDL cholesterol than *GSTM1-active/GSTT1-active* (data not shown). Supplementary ***Table*** ***1*****.**

## Discussion

**Fig. 1:**
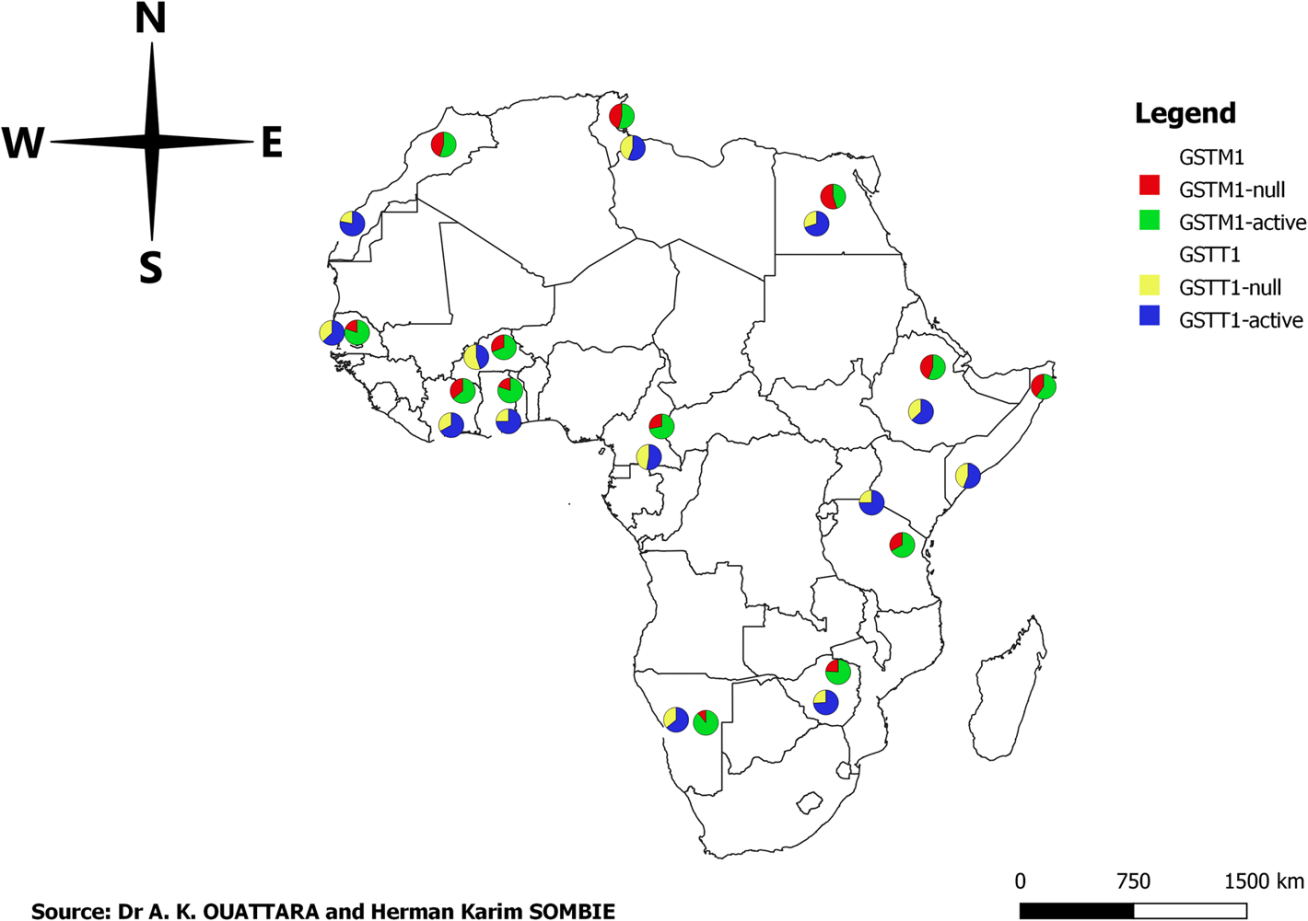
GSTM1 and GSTT1 genes deletion frequency in African countries [37–46]. **Legend***: Pie charts show frequency of GSTM1-null (red) and GSTT1-null (yellow) frequency in each country*

There is increasing interest in the role of *Glutathione S-transferase* polymorphism as a contributory factor to oxidative stress and consequently to cellular damage and physiological anomalies such us cancers, diabetes, cardiovascular disorders [36]. The present study aimed to characterize firstly the null genotype of *GSTM1* and *GSTT1* genes in Burkina Faso and secondly to analyzes a possible association between them and the risk of developing essential hypertension. Our study showed in the control group that the frequency of *GSTM1-null* and *GSTT1-null* were 31.23 and 55.76% respectively. There were several studies about *GSTM1* and *GSTT1* deletion and some diseases process, so Fig. 1 summarizes the frequencies of the *GSTM1* and *GSTT1* deletion in some African countries [37–46].

In our study we analyzed also the relationship between Glutathione S-transferase M1 and T1 genes deletion and their connection with essential hypertension and any complications that may have accompanied this disease in Burkina Faso. Association between *GSTM1* and *GSTT1* polymorphisms and cardiovascular disorders has long been studied. Some studies have shown that *GSTM1* [25, 26, 47, 48] and/or *GSTT1* [26, 27, 48–51] were associated with essential hypertension risk, other authors have shown that only the double deletion *GSTM1/GSTT1* was associated with essential hypertension risk, and other failed to confirm any association [29]. The Table 5 summarize previous associations studies of *GSTM1* and *GSTT1* with risk of hypertension, specifies the size and type of population as well as the type of PCR and the main results obtained. In the present study, we showed that the frequency of *GSTT1-null* genotype was significantly higher in essential hypertension group than control group, indicating a possible association between *GSTT1* and risk of developing essential hypertension with a power of more than 85%. We failed to replicate association of essential hypertension with *GSTM1*-*null* genotype and the double deletion *GSTM1-null*/*GSTT1-null* even if *GSTM1*-null frequencies only give us a power of less than 40%.

**Table 5:** Summary of previous studies examining *GSTM1* and *GSTT1* polymorphisms and hypertension risk

Country	Population (Cases/controls)	Ethnicity	Genotyping method	association with the risk of hypertension	Years	First author/ references
Portugal	Hypertension/congestive heart failure (94/207)	Caucasian	PCR	*GSTT1*-active	2007	Marinho [49]
Egypt	General population (40/40)	African	Multiplex PCR	*GSTM1*-null/*GSTT1*-null	2009	Bessa [24]
Italy	Older subjects (255/99)	Caucasian	PCR	*GSTM1*-null	2009	Capoluongo [25]
India	Tea garden workers (223/236)	Asian	Multiplex PCR	*GSTM1*-null; *GSTT1*-null (in smokers)	2011	Borah [26]
Italy	General population (193/210)	Caucasian	Multiplex PCR	*GSTT1*-null (women)	2011	Polimanti [27]
Korea	General population (227/130)	Asian	PCR	*GSTM1*-null	2011	Han [47]
United Arab Emirates	General population (30/33)	Asian	Multiplex PCR	none	2012	Hussain [29]
Korea	Lead-exposed workers (258/497)	Asian	Multiplex PCR	*GSTT1*-active	2012	Lee [50]
Slovenia	hypertension/type 2 diabetes (1015)	Caucasian	Multiplex PCR	*GSTM1*-null *GSTT1*-null	2014	Petrovic [51]
India	General population (138/116)	Asian	Multiplex PCR	*GSTT1*-null; *GSTM1-*active	2015	Abbas [48]

Essential hypertension is a complex disease, with many risk factors [52], and there is growing evidence that gene interactions with environmental factors may increase susceptibility to essential hypertension [53], so that certain genetic variants have only a significant effect in specific populations. In our study we found that alcohol use, lack of sport, overweight, obesity, central obesity, and family history of hypertension were factors that increased the risk of developing essential hypertension in our study population. In addition, we performed a stratified analysis by BMI, alcohol use and smoking status of the association between *GSTM1*, *GSTT1* variants and essential hypertension; The results showed that association between *GSTT1*-null and essential hypertension seems to be significant especially in alcohol users but independent of obesity and smoking even if some of the comparisons do not have enough samples. The present work confirms that the deletion of *GSTT1* may increase the risk of developing essential hypertension, and this is partly related to the antioxidant activity of the GST enzyme. In fact, Nitric oxide (released by the endothelium) plays a major role in arterial relaxation and is rapidly degraded by the oxygen-derived free radical superoxide anion [54], the presence of which largely depends on the activity of the GST enzyme. Therefore, GST variants may influence nitric oxide bioavailability and subsequently may influence the individual susceptibility to essential hypertension.

In addition, we also compared in hypertensive group, means of certain cardiovascular risk markers between null and active genotypes of *GSTM1* and *GSTT1* genes. We found significantly lower triglyceride in *GSTM1*-null genotype compared to *GSTM1*-active; we also observed in *GSTT1*-null subjects, significantly higher waist circumference and serum HDL cholesterol compared to *GSTT1*-active genotype. The double deletion *GSTM1-null*/*GSTT1-null* was associated with body mass index, waist circumference and serum HDL cholesterol increasing. These results suggest a probable association of Glutathione S-transferase M1 and T1 genes deletion with body fat and serum cholesterol level. There is not a lot of study about these association, while Almoshabek *and al.* reported in young age Saudis that frequencies of *GSTM1-active/GSTT1-null* (OR = 2.70; *p* <  0.001) and *GSTM1-null/GSTT1-null* (OR = 2.43, *p* = 0.018) were significantly higher in overweight/obese as compared to normal weight [55]. Our results are in accordance with those of Afrand *and al.* in Zoroastrian females (Iran), that reported significantly lower HDL cholesterol in *GSTT1*-null as compared to *GSTT1*-active genotype in metabolic syndrome [56], and Khalilzadeh *and al.* that reported higher levels of triglyceride, fasting blood sugar, total cholesterol, LDL cholesterol, body mass index and HDL cholesterol in patients with type 2 diabetes with *GSTT1*-null genotype than in those with the *GSTT1*-active genotypes [57].

## Conclusion

In conclusion, our study suggests the significant association between *GSTT1-null* genotype and the risk of developing essential hypertension in Burkinabe population, therefore the important role of oxidative stress in the development of essential hypertension. The study also suggests that the double deletion *GSTT1-null*/*GSTM1-null* may affect body lipids repartition in hypertensive. However large-scale study will be necessary to fully comprehend the role of *GSTM1* and *GSTT1* variant in the development of essential hypertension.

## Supplementary information

**Additional file 1: Fig. S1.** Locations of *GSTM1*, *GSTT1* and *β-globin* genes and corresponding bands in electrophoresis gel. This file shows locations of *GSTM1*, *GSTT1* and *β-globin* genes on chromosomes and corresponding bands. The number 1 through 19 represents individual sample and M represents Molecular weight marker. The strategy to identify presence or absence of *GSTM1*or *GSTT1*was as followed: to validate a PCR product (corresponding to a sample), we must have a band corresponding to *β-globin* and presence or absence of *GSTM1* or *GSTT1* was indicated respectively by the presence or absence of bands corresponding for each gene.

**Additional file 2: Table S1.** Distribution of cardiovascular risk markers according to *GSTM1* and *GSTT1* variants in hypertensive group. This ***table*** show and compare average of cardiovascular risk markers such as BMI, WC, serum level of blood sugar, TC, HDL-c, LDL-c and Triglycerides according to *GSTM1* and *GSTT1* variants in hypertensive group.

**Additional file 3 Data collection.** Information sheet and questionnaire. This file shows the fact sheets used to explain the study during the recruitment of the participants and the questionnaire which served for the data collection.

## Data Availability

The dataset generated in this study is available from NCBI Nucleotide under the accession number LC517160.1.

## Abbreviations

BMI: Body mass index
CERBA: Pietro annigoni biomolecular research center
DBP: Diastolic blood pressure
EDTA: Ethylenediaminetetraacetic
*GSTA*: Glutathione S-transferases alpha
*GSTK*: Glutathione S-transferases kappa
*GSTM1*: Glutathione S-transferases mu 1
*GSTO*: Glutathione S-transferases omega
*GSTP*: Glutathione S-transferases pi
*GSTS*: Glutathione S-transferases sigma
*GSTT1*: Glutathione S-transferases theta 1
*GSTZ*: Glutathione S-transferases zeta
HDL-c: High-density lipoprotein cholesterol
LABIOGENE: Laboratory of molecular biology and genetics
LDL-c: Low-density lipoprotein cholesterol
MD: Means difference
PCR: Polymerase chain reaction
ROS: Reactive Oxygen Species
SBP: Systolic blood pressure
SD: Standard deviation
SPSS: Statistical package for the social sciences
TC: Total cholesterol
WC: Waist circumference
