# Supplementary material for: Glutathione S-transferase M1 and T1 genes deletion polymorphisms and risk of developing essential hypertension: a case-control study in Burkina Faso population (West Africa)
Source: BMC Med Genet. 2020 Mar 19;21:55. doi: 10.1186/s12881-020-0990-9 (PMC7081581; doi:10.1186/s12881-020-0990-9)
Supplement: Supplementary file 1 — Additional file 1: Fig. S1. Locations of GSTM1, GSTT1 and β-globin genes and corresponding bands in electrophoresis gel. This file shows locations of GSTM1, GSTT1 and β-globin genes on chromosomes and corresponding bands. The number 1 through 19 represents individual sample and M represents Molecular weight marker. The strategy to identify presence or absence of GSTM1or GSTT1was as followed: to validate a PCR product (corresponding to a sample), we must have a band corresponding to β-globin and presence or absence of GSTM1 or GSTT1 was indicated respectively by the presence or absence of bands corresponding for each gene. [file 12881_2020_990_MOESM1_ESM.docx]

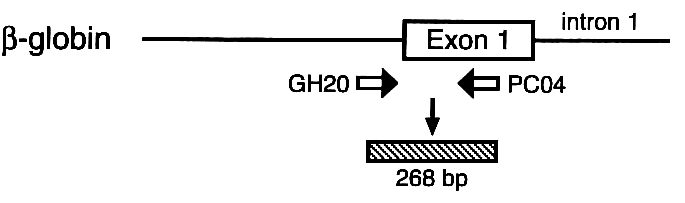

[22q11.23](http://atlasgeneticsoncology.org/Bands/22q11.html)

11p15.4

[1p13.3](http://atlasgeneticsoncology.org/Bands/1p13.html)

480 bp

268 bp

219 bp

***M****= Molecular weight marker (100bp); GSTM1-active/GSTT1-active=* ***6, 9, 11, 16****; GSTM1-active/GSTT1-null=* ***1, 2, 3, 4, 12, 13, 18, 19****;* *GSTM1-null/GSTT1-active=* ***14, 15, 17****; GSTM1-null/GSTT1 null=* ***5, 7, 8, 10.***

**Figure S1:** Locations of *GSTM1*, *GSTT1*, *β-globin* genes and corresponding bands in electrophoresis gel. Adapted from Chen *and al.* [35].
