# Supplementary material for: Glutathione S-transferase M1 and T1 genes deletion polymorphisms and risk of developing essential hypertension: a case-control study in Burkina Faso population (West Africa)
Source: BMC Med Genet. 2020 Mar 19;21:55. doi: 10.1186/s12881-020-0990-9 (PMC7081581; doi:10.1186/s12881-020-0990-9)
Supplement: Supplementary file 2 — Additional file 2: Table S1. Distribution of cardiovascular risk markers according to GSTM1 and GSTT1 variants in hypertensive group. This table show and compare average of cardiovascular risk markers such as BMI, WC, serum level of blood sugar, TC, HDL-c, LDL-c and Triglycerides according to GSTM1 and GSTT1 variants in hypertensive group. [file 12881_2020_990_MOESM2_ESM.docx]

**Table S1:** Distribution of cardiovascular risk markers according to *GSTM1* and *GSTT1* genotypes in hypertensive group.

| Genotypes | BMI (Kg/m^2^) | WC (cm) | TC (mM) | HDL-c (mM) | LDL-c (mM) | triglycerides (mM) |
| --- | --- | --- | --- | --- | --- | --- |
| *^#^ GSTM1-positive* (+) | 28.22 ± 6.67 | 96.53 ± 12.00 | 5.12 ± 1.14 | 1.41 ± 0.63 | 3.07 ± 1.13 | 1.29 ± 0.78 |
| *GSTM1-null* (-) | 28.72 ± 5.56 | 95.58 ± 13.07 | 5.03 ± 1.25 | 1.33 ± 0.59 | 2.95 ± 0.96 | 1.02 ± 0.54 |
| *p* value | 0.17 | 0.06 | 0.57 | 0.48 | 0.43 | 0.02 |
| *^#^ GSTT1-positive* (+) | 28.09 ± 6.48 | 94.88 ± 12.79 | 5.00 ± 1.32 | 1.14 ± 0.36 | 3.10 ± 0.98 | 1.20 ± 0.52 |
| *GSTT1-null* (-) | 28.46 ± 6.39 | 96.91 ± 12.00 | 5.13 ± 1.10 | 1.49 ± 0.67 | 3.03 ± 1.13 | 1.24 ± 0.81 |
| *p* value | 0.29 | < 0.001* | 0.38 | 0.001* | 0.62 | 0.73 |
| *^#^ GSTM1*(*+*) */GSTT1*(*+*) | 28.90 ± 7.33 | 96.64 ± 12.70 | 5.12 ± 1.21 | 1.19 ± 0.40 | 3.20 ± 1.00 | 1.31 ± 0.52 |
| *GSTM1*(*+*) */ GSTT1*(*-*) | 28.00 ± 6.46 | 96.49 ± 11.81 | 5.12 ± 1.13 | 1.47 ± 0.67 | 3.04 ± 1.16 | 1.29 ± 0.83 |
| *GSTM1*(*-*) */ GSTT1*(*+*) | 26.89 ± 4.79 | 92.23 ± 12.67 | 4.85 ± 1.48 | 1.07 ± 0.32 | 2.97 ± 0.97 | 1.05 ± 0.50 |
| *GSTM1*(*-*) */ GSTT1*(*-*) | 30.44 ± 5.76 | 98.71 ± 12.84 | 5.20 ± 0.99 | 1.60 ± 0.68 | 2.94 ± 0.99 | 0.98 ± 0.60 |
| *p* value (+/+) *vs* (-/-) | 0.01* | 0.01* | 0.74 | 0.01* | 0.26 | 0.05 |

*Values are reported as means ± standard deviation; Statistical analysis (active versus null) by t test; #: reference; *: significant p value; SBP: systolic blood pressure; DBP: diastolic blood pressure; WC: waist circumference; HDL-c: high density lipoprotein cholesterol; LDL-c: low density lipoprotein cholesterol; TC: total cholesterol; vs: versus.*
